# Ceramide-Induced Cell Death Depends on Calcium and Caspase-Like Activity in Rice

**DOI:** 10.3389/fpls.2020.00145

**Published:** 2020-02-26

**Authors:** Quan-Fang Zhang, Jian Li, Fang-Cheng Bi, Zhe Liu, Zhen-Yi Chang, Ling-Yan Wang, Li-Qun Huang, Nan Yao

**Affiliations:** State Key Laboratory of Biocontrol, Guangdong Provincial Key Laboratory of Plant Resources, School of Life Sciences, Sun Yat-sen University, Guangzhou, China

**Keywords:** calcium, caspase, ceramide, programmed cell death, rice

## Abstract

Ceramide sphingolipids are major components of membranes. C2 and C6 ceramides induce programmed cell death (PCD) in animals and plants, and we previously showed that C2 and C6 ceramides induce PCD in rice (*Oryza sativa*) protoplasts. However, the mechanistic link between sphingolipids and PCD in rice remains unclear. Here, we observed that calcium levels increased rapidly after ceramide treatment. Moreover, the calcium channel inhibitor LaCl_3_ and the intracellular calcium chelator acetoxymethyl-1, 2-bis (2-aminophenoxy) ethic acid (BAPTA-AM) inhibited this calcium increase and prevented ceramide-induced PCD. Moreover, caspase-3-like protease activity increased significantly in C6 ceramide-treated protoplasts, and a caspase-specific inhibitor prevented C6 ceramide-induced cell death. We also detected the other typical PCD events including ATP loss. DIDS (4, 49-diisothiocyanatostilbene- 2, 29-disulfonic acid), an inhibitor of voltage-dependent anion channels (VDACs), decreased C6 ceramide-induced cell death. Together, this evidence suggests that mitochondria played an important role in C6 ceramide-induced PCD.

## Introduction

Sphingolipids are present in all higher organisms, and ceramide is a key compound in sphingolipid metabolism (Hannun and Obeid, 2018). Ceramide mediates a number of biological processes in animals, including proliferation, differentiation, growth arrest, inflammation, heat stress responses, and cell death (Ogretmen, 2018). Programmed cell death (PCD) is a physiological, genetically controlled form of cell death, characterized by condensation of the cytoplasm, nuclear DNA cleavage into oligonucleosome-sized fragments, and chromatin condensation (Hengartner, 2000). In mammals the permeability transition pore (PTP), a high-conductance inner membrane channel activated by increased matrix Ca^2+^ and oxidative stress, functions as part of the PCD signaling cascade (Carraro and Bernardi, 2016). PCD is involved in defense, development, and the response to stress (Reape and McCabe, 2010). In mammalian cells, a close association between the production of ceramide and the onset of apoptosis/PCD has been well established (Hannun and Obeid, 2008).

Numerous reports have evaluated the role of sphingolipids in regulating apoptosis-like PCD in plants (Alden et al., 2011; Berkey et al., 2012) and demonstrated that the disruption of sphingolipid metabolism leads to abnormal plant development, even lethality. In *Arabidopsis thaliana*, the LAG1 HOMOLOG 1 (LOH1), LOH2, and LOH3 enzymes are responsible for the synthesis of ceramide, and the *loh1* mutant shows spontaneous cell death under short-day conditions. The *loh1 loh3* mutant plants contain no very-long-chain fatty acid sphingolipids and die in early development (Markham et al., 2011). The ceramide kinase deficient mutant *accelerated cell death 5* (*acd5*) displays spontaneous cell death and accumulates high levels of ceramides (Bi et al., 2014). Similar phenotypes were observed in the GlcCer synthase (GCS) mutant *gcs-1* (Msanne et al., 2015). However, the mechanistic link between sphingolipids and PCD in rice remains unclear.

Reactive oxygen species (ROS) play an important role in PCD induced by sphingolipids. In animals, C16-ceramide, sphingosine, and sphinganine directly inhibit the activity of mitochondrial complex IV, leading to ROS production and oxidative stress (Zigdon et al., 2013). Free sphingobases t18:0, d18:0, and d17:1, but not d20:0, trigger ROS and cell death in plants, a process that requires respiratory burst oxidase homolog D (RbohD) for early ROS induction (Peer et al., 2011). In addition, the production of endogenous ROS is often affected by intracellular calcium ion (Ca^2+^) concentration. Variation of intracellular Ca^2+^/calmodulin (CaM) concentration triggers PCD in plants (Li et al., 2018). However, whether ceramides induce PCD through ROS or Ca^2+^ signaling in rice remains unclear.

Release of Cytochrome *c* (Cyt *c*) from mitochondria into the cytosol is a typical characteristic of PCD in animals, and work in mammals showed that released Cyt *c* forms a complex with Apaf-1, dATP, and pro-caspase 9 to activate downstream apoptotic factors (Xiao D. et al., 2018). However, in plants, direct structural homologs of animal caspases with an analogous cleavage specificity and function have not been identified, although some specific peptide inhibitors of animal caspases have been shown to affect the development of plant PCD (Bonneau et al., 2008). In fact, Cyt *c* release from the mitochondria occurs in numerous reports (yet not all), such as plant PCD (Li et al., 2017). We previously showed that ceramide-induced Cyt *c* release occurred before protoplast cell death in Arabidopsis (Yao et al., 2004). Whether the release of Cyt *c* occurs in rice PCD was still unknown.

Here, we investigate the features of sphingolipid induced-PCD, using C2/C6-ceramide. These synthetic, short-chain ceramides cross the cell membrane and simulate the accumulation of ceramide in the cell during apoptosis in plant and animal cells (Yao et al., 2004; Hernández-Corbacho et al., 2015). Previous studies used C2/C6-ceramide to examine ceramide-mediated PCD in plant cells (Yao et al., 2004; Townley et al., 2005; Bi et al., 2011), and we report that calcium and caspase-like are involved in rice protoplast cell death induced by ceramides. Moreover, ceramides induced mitochondrial dysfunction but not Cyt *c* release.

## Materials and Methods

### Plants and Materials

Rice plants (*Oryza sativa* ssp. *japonica* cv. Nipponbare) were grown in water and incubated at room temperature in the dark. Rice protoplasts were isolated from 10-day-old seedlings as described (Shen et al., 2010; Bi et al., 2011). Briefly, rice seeds were germinated on half-strength Murashige and Skoog (½ MS) medium under light for 3 days. Seedlings were then cultured on ½ MS medium in the dark at 26°C for 10 days. We stripped the coleoptiles, cut etiolated young seedlings into approximately 0.5-mm strips and placed these in baffled flasks containing 0.6 M mannitol for 10 min. The chopped tissues were then transferred to an enzyme mixture [1.5% (w/v) cellulase RS and 0.75% (w/v) macerozyme R10 (Kinki Yakult, Tokyo, Japan), 10 mM MES (pH 5.7), 0.1% (w/v) BSA, 1 mM CaCl_2_, 5 mM β-mercaptoethanol and 0.6 M mannitol] and shaken at low speed at room temperature for 3–4 h. Protoplasts were collected with a 40 μm nylon mesh and washed in W5 solution (154 mM NaCl, 125 mM CaCl_2_, 5 mM KCl, 2 mM MES, 5 mM glucose adjusted to pH 5.7 with KOH). The viability of protoplasts after treatment was determined using fluorescein diacetate (FDA) staining with a hemacytometer and a fluorescence microscope with Zeiss filter set 38 (Axio Imager A1, Carl Zeiss).

*N*-acetyl-D-erythro-sphingosine (C2-ceramide, 1901), *N*-hexanoyl-D-sphingosine (C6-ceramide, 1809), N-acetyl-D-erythro-dihydrosphingosine (C2-dihydroceramide, 1834), and N-hexanoyl-D-erythro-dihydrosphingosine (C6-dihydroceramide, 1910) were purchased from Matreya (Pleasant GAP, PA, USA), and dissolved in ethanol. We made stock solutions for C2-ceramide (50 mM) and C6-ceramide or C6-dihydroceramide (C6-DHC, 100 mM) in ethanol. The final concentrations used for treatments were 50 μM for C2-ceramide and 100 μM for C6-ceramide or C6-DHC.

The anion channel blocker DIDS (4, 4-diisothiocyanatostilbene-2, 2-disulfonic acid, D3514), free radical scavenger NAC (N-acetylcysteine, A0737), calcium channel blocker lanthanum chloride (LaCl_3_, SML0902), permeable intracellular calcium chelator acetoxymethyl-1, 2-bis (2-aminophenoxy) ethic acid (BAPTA-AM, A1076), calcium ionophore A23187 (C9275), protease inhibitors pepstatin A (P5318), leupeptin (L2884), protease inhibitor cocktail (P8340), acid-washed glass beads (G8772), and fluorescein diacetate (FDA, F7378) were obtained from Sigma-Aldrich.

MitoTracker Red CMXRos (CMXRos, M7512), 5-and-6-chloromethyl-2, 7-dichlorodihydro- fluorescein diacetate, acetyl ester (CM-H_2_DCFDA, C6827), 4-amino-5-methylamino-2, 7-difluorofluorescein diacetate (DAF-FM, D23842), Hoechst33342 (H21492), ATP assay kit (A22066), and the calcium indicator Fluo-4/AM (F23917) were obtained from Invitrogen. The fluorogenic substrates for caspase-1 (Ac-YVAD-AMC, 149231-65-2) and caspase-3 (Ac-DEVD-AMC, 169332-61-0) were purchased from BIOMOL. Anti-VDAC1 polyclonal antibody (AS07212, Agrisera, Sweden) was used for western blotting as a mitochondrial marker.

### Intracellular Calcium Detection

For flow cytometry, rice protoplasts were incubated with ceramide or the indicated treatments for 15–120 min and then loaded with 2 μM fluo-4/AM by incubation for 15 min at room temperature in the dark. Cells were washed twice with W5 solution (154 mM NaCl 125mM CaCl_2_, 5 mM KCl, 5 mM glucose, 2 mM MES, adjusted pH to 5.7 with 1 M KOH), resuspended, and then subjected to flow cytometry (BD Biosciences, FACSCalibur, San Diego, CA, USA) using excitation with a single 488 nm argon laser. At least 30,000 cells were collected per sample. The experiments were repeated at least three times with similar results. Data analysis used the CellQuest software (BD FACS Calibur, Becton Dickinson) and WinMDI2.9 software (Scripps Research Institute, San Diego, CA).

### ROS Detection

Intercellular ROS production was measured by monitoring the fluorescence of CM-H_2_DCFDA (Yao and Greenberg, 2006). The treated protoplasts were collected and loaded with the red fluorescent mitochondrial label “MitoTracker^®^ Red” CMXRos (50 nM) for 5 min, then 5 nM CM-H2DCFDA for 10 min in the dark; after being washed in W5 medium, the samples were observed using 488 nm excitation (emission: 498 to 532 nm). The ﬂuorescence signals were observed by confocal microscopy. ROS signals were also detected by flow cytometry as described above.

### Caspase-Like Activity Assays

Control and C6-treated protoplasts were collected and homogenized in extraction buffer as described (Qu et al., 2009). Briefly, samples were homogenized and centrifuged, then the supernatants were collected. The supernatant samples (15 μg protein) were then mixed with 100 μM of the indicated fluorogenic substrate (Ac-YVAD-AMC or Ac-DEVD-AMC) and caspase assay buffer was added to a final volume of 200 μl. Release of the AMC fluorophore hydrolyzed from the peptide substrate was quantified at 460 nm (excitation 380 nm) by using a fluorescence microplate reader (BioTek Synergy2, USA) after incubation at 30°C for 30 min. Each assay was carried out in at least three independent repeats. For inhibitor assays, protease inhibitors (200 μM pepstatin A or 100 μM leupeptin) and caspase-specific inhibitors (Ac-YVAD-CHO and Ac-DEVD-CHO) were used.

### Measurement of Intracellular ATP

Intracellular ATP was measured using an ATP determination kit (A22066, Molecular Probes, Invitrogen) according to the manufacturer’s instructions (Molecular Probes, Invitrogen), and a previous report (Krause and Durner, 2004). Briefly, control and C6-treated cells were harvested and washed in 0.1 M PBS (pH 7.4). After the sample was centrifuged at 1,500 rpm at 4°C for 30 min, the pellet was resuspended in 200 μl buffer [100 mM Tris-HCL (pH 7.75), 4 mM EDTA] and boiled for 10 min. After centrifugation, the supernatant was added to a polystyrene round-bottom tube (BD Biosciences) containing 90 μl of luciferase reaction solution and gently mixed. The luminescence was measured using a luminometer (emission maximum ~560 nm at pH 7.8, Lumat LB 9507, Berthold, Germany). Standard curves were prepared in all experiments with various ATP concentrations, and calculations were made using the standard curve.

### Subcellular Fractionation and Western Blotting

Mitochondrial and cytosolic fractions were isolated from protoplasts as described by Yao et al. (2004) with minor modifications. Briefly, control and C6-treated protoplasts were collected and washed with W5 (154 mM NaCl, 125 mM CaCl_2_, 5 mM KCl, 5 mM glucose, 2 mM MES, pH5.7). The protoplast samples were collected and homogenized in buffer (50 mM Tris-HCl, pH 7.5, 10 mM MgCl_2_, 1 mM CaCl_2_, 1 mM EDTA, 0.25 M sucrose, 1 mM DTT). The homogenate was filtered through two layers of Miracloth (Calbiochem, Darmstadt, Germany). Cellular debris was pelleted by centrifugation at 1,000 *g* for 10 min. The supernatant was spun at 10,000 *g* for 10 min. The supernatant was used as the cytosolic protein fraction. The pellet fraction was used as the fraction enriched in mitochondria.

Mitochondrial fractions were incubated with protein exaction buffer [50 mM HEPES, pH 7.4, 3 mM DTT, 0.1 mM EDTA, 2‰ protease inhibitor cocktail (4693159001, Roche)] for 10 min at 4°C, and samples were centrifuged at 12,000 *g* for 10 min to remove insoluble material. Supernatants, considered the soluble mitochondrial fraction, were used for the western blot as described (Bi et al., 2014). Rabbit polyclonal anti-VDAC-1 was used as a mitochondrial marker (Agrisera, Vännäs, Sweden).

### Ultrastructural Analysis and Immunolocalization

For conventional transmission electron microscopy, 6 × 10^6^ protoplasts were treated with C6 ceramide and fixed and embedded as described previously (Bi et al., 2014). For immuno-electron microscopy, the procedure was performed as described (Bi et al., 2014) with minor modifications. Briefly, protoplasts were pre-fixed with 4% (v/v) paraformaldehyde and 0.5% (v/v) glutaraldehyde, and post-fixed with 0.1% (w/v) osmium tetroxide. Samples were dehydrated with ethanol and embedded in K4M (Lowicryl K4M Polar Kit, Polyscience). Ultrathin sections were cut and collected on nickel grids. Samples were incubated with antibodies (primary antibody diluted 1:50 for cytochrome *c*) after blocking with BSA-TBST buffer (150 mM NaCl, 50 mM Tris, 0.5% BSA, pH 7.4). Antibodies were incubated with the samples for 2 h at room temperature. Goat-antirabbit IgG labeled with 10-nm colloidal gold (Electron Microscopy Science) was used to detect the primary antibodies. Sections were stained with uranyl acetate and lead citrate, then observed under a transmission electron microscope (JEOL JEM-1400) operated at 120 kV.

### Sphingolipid Analysis

Sphingolipids were extracted based on a previous description (Li et al., 2016). Measurement of sphingolipids was performed and the data were analyzed with a Shimadzu UFLC-XR (Shimadzu, Japan) coupled with a hybrid quadrupole time-of-ﬂight mass spectrometer (AB SCIEX Triple TOF 5600+, Foster City, CA, USA). The components of sphingolipids were determined as described previously (Bi et al., 2014).

### Statistical Analysis

All experiments were repeated at least three times. Statistical analyses were performed using Statview statistical package 5.0.1 (SAS Institute, Cary, North Carolina, USA) for Macintosh as described previously (Shen et al., 2010).

## Results

### PCD Occurred After C6 Treatment in Rice

**Figure 1:**
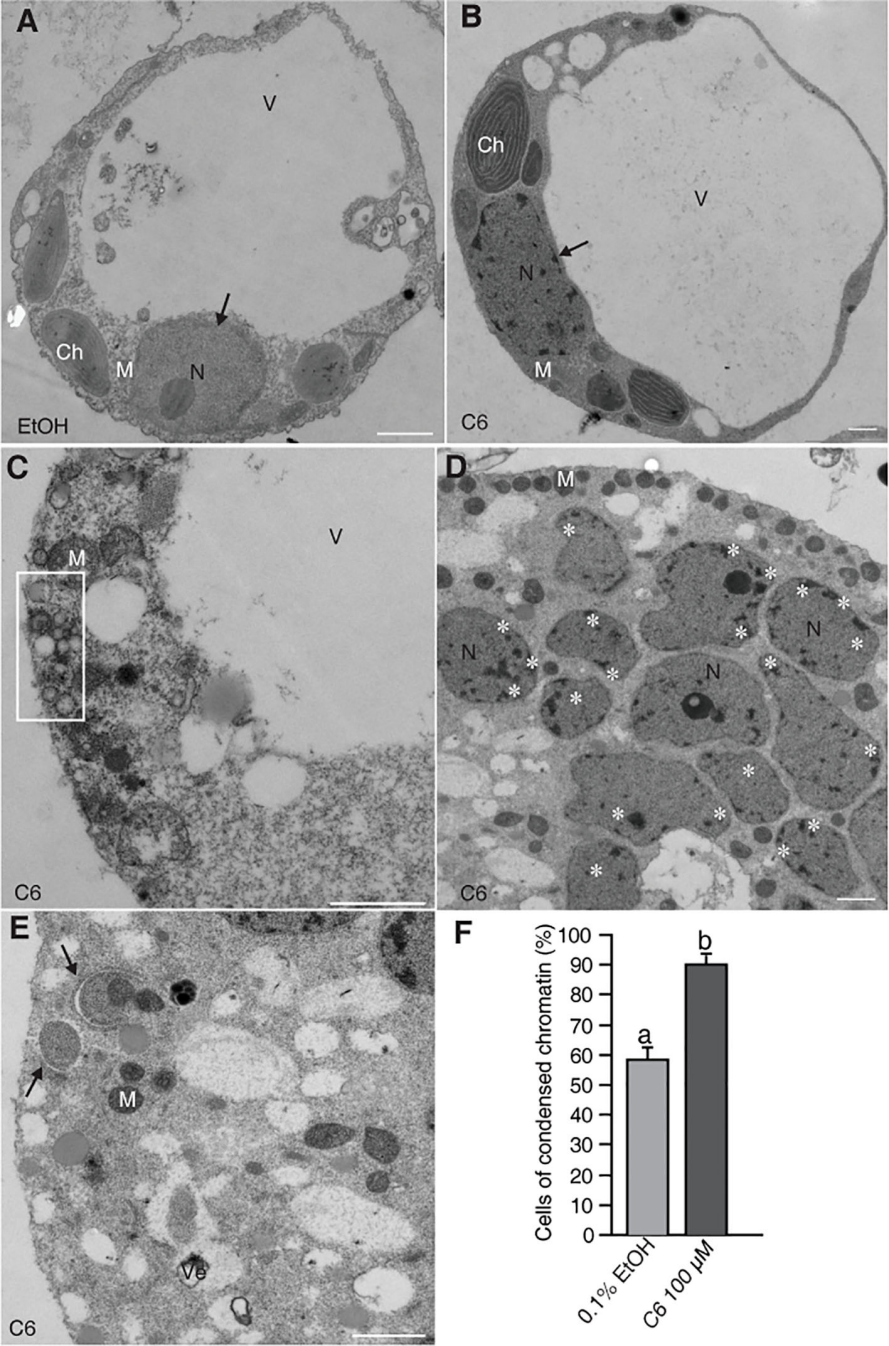
Electron micrographs of rice protoplasts after C6 ceramide treatments. Rice protoplasts were incubated with solvent control (0.1% EtOH) or 100 µM C6 ceramide for indicated times and then fixed for electron microscopy observation. **(A)** A control cell. Note a normal vacuole and nucleus with nucleolus (arrow). **(B)** A C6 ceramide-treated cell. Note condensed chromatin in the nucleus (arrow) and relatively normal morphology of organelles. **(C)** A dying C6 ceramide-treated cell. Note abnormal mitochondria and vesicle clusters along plasma membrane (square). **(D)** A 36 h C6 ceramide-treated cell. Note numerous nuclear fragmented nuclei with condensed chromatin (white stars). **(E)** A 36 h C6 ceramide-treated cell. Note double-membrane bound autophagic vacuole (autophagosome) sequestering mitochondria (arrow). **(F)** The frequency of nuclei with condensed chromatin. A total of 20 cells were included for each treatment. This experiment was repeated three times using independent samples. Letters indicate that values differed based on Fisher’s protected least significant difference (PLSD) test, a *post hoc* multiple *t* test (*p* < 0.05). Error bars indicate standard deviations. Ch, chloroplast; M, mitochondrion; N, nucleus; V, vacuole; Ve, vesicle cluster. Bars in **(A–E)** = 1 µm.

Previously, we reported detection of DNA laddering, indicative of DNA fragmentation during PCD, in rice protoplasts after 12–24 h of C6 treatment (Bi et al., 2011). Here, we fixed the protoplasts after C6 treatment and observed ultrastructural changes by electron microscopy. We found normal cell morphology in control cells at 24 h (**Figure 1A**) and condensed chromatin in C6-treated protoplasts for 24 h (**Figure 1B**). We also observed many small bubbles around the plasma membrane in cells treated with C6 for 24 h (**Figure 1C**). At 36 h of C6 treatment, fragmented nuclei (**Figure 1D**) and mitochondria with double membranes were observed (**Figure 1E**). The frequency of nuclei with apparently condensed chromatin in cells treated with C6 for 36 h was significantly higher in C6-treated cells than in control treatments (**Figure 1F**).

**Figure 2:**
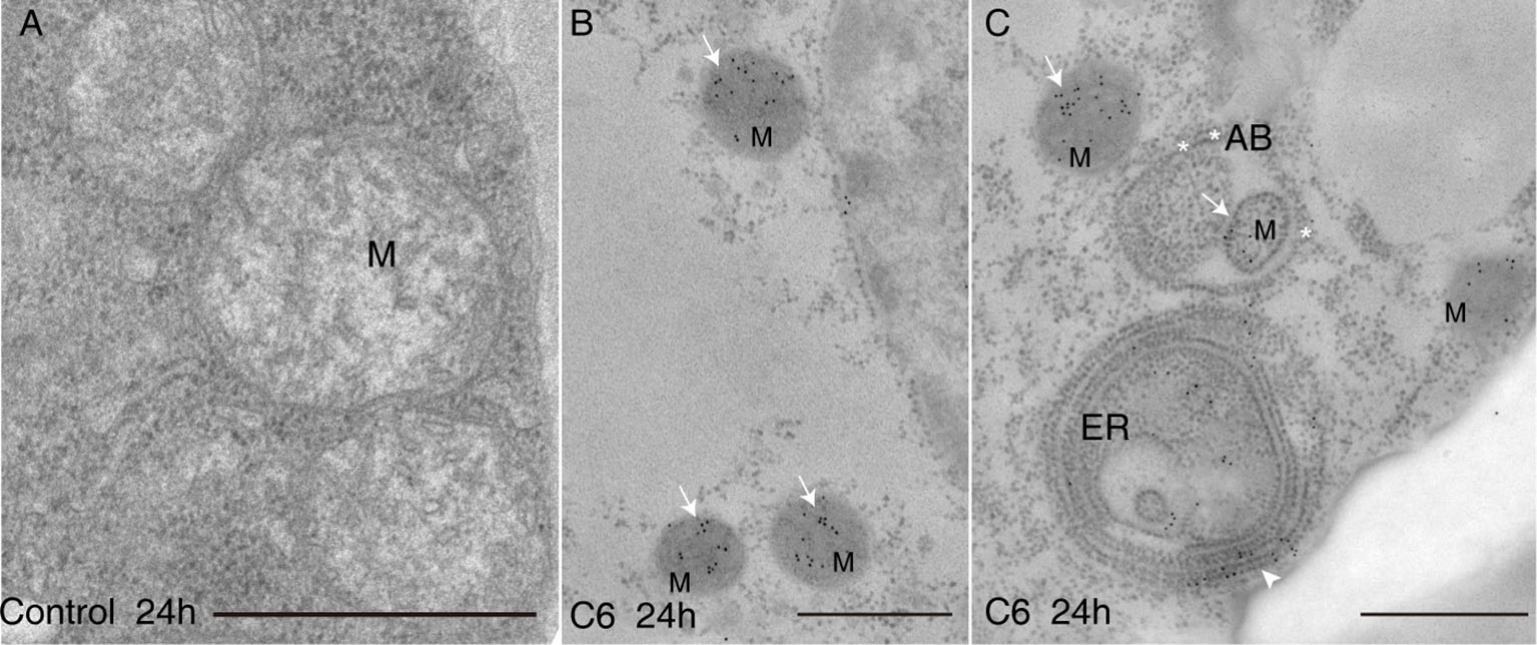
Ceramides accumulated in mitochondria after C6 ceramide treatment. Immunolocalization of ceramide in cells treated with 0.1% EtOH **(A)** or 100 μM C6 ceramide for 24 h **(B**, **C)** by using anti-ceramide antibodies. Note immuno-gold particles on mitochondria (white solid arrows in **B** and **C**) and endoplasmic reticulum (**C**, white arrow). Mitochondria are packaged into autophagic bodies (AB) in the C6 ceramide-treated cell (**C**, white star). ER, endoplasmic reticulum; Mt, mitochondrion; AB, autophagic body; Bar=500 nm.

Because C6 ceramide treatment markedly increased cell death, we examined the effect of C6 on the accumulation of intracellular ceramides. Measuring the levels of intracellular ceramides showed that long chain bases (LCBs) and ceramide contents increased substantially after C6 treatments (**Supplemental Figure 1A**). When we compared ceramides with LCB moieties, we found that d18:1 ceramides increased the most in C6 ceramide-treated cells (**Supplemental Figure 1B** **-**top). By comparing ceramides with different fatty acid moieties, we found ceramides containing long chain fatty acids (C16) as the major component in cells treated with C6 ceramide (**Supplemental Figure S1B** **-**bottom). We also used sphingosine to treat protoplasts for 4 h, and found that cell viability rapidly decreased (**Supplemental Figure 1C**). We next observed intracellular ceramide localization after C6 ceramide treatment by immuno-electron microscopy with anti-ceramide antibodies. The accumulated ceramides mostly targeted mitochondria and the endoplasmic reticulum (**Figures 2B, C**). Moreover, we observed mitochondria that accumulated ceramides and were wrapped by an autophagosome membrane (**Figure 2C**). These data indicate that C6 upregulates endogenous ceramide levels.

### Calcium Signaling Was Involved in C6 Ceramide-Induced PCD in Rice Protoplasts

**Figure 3:**
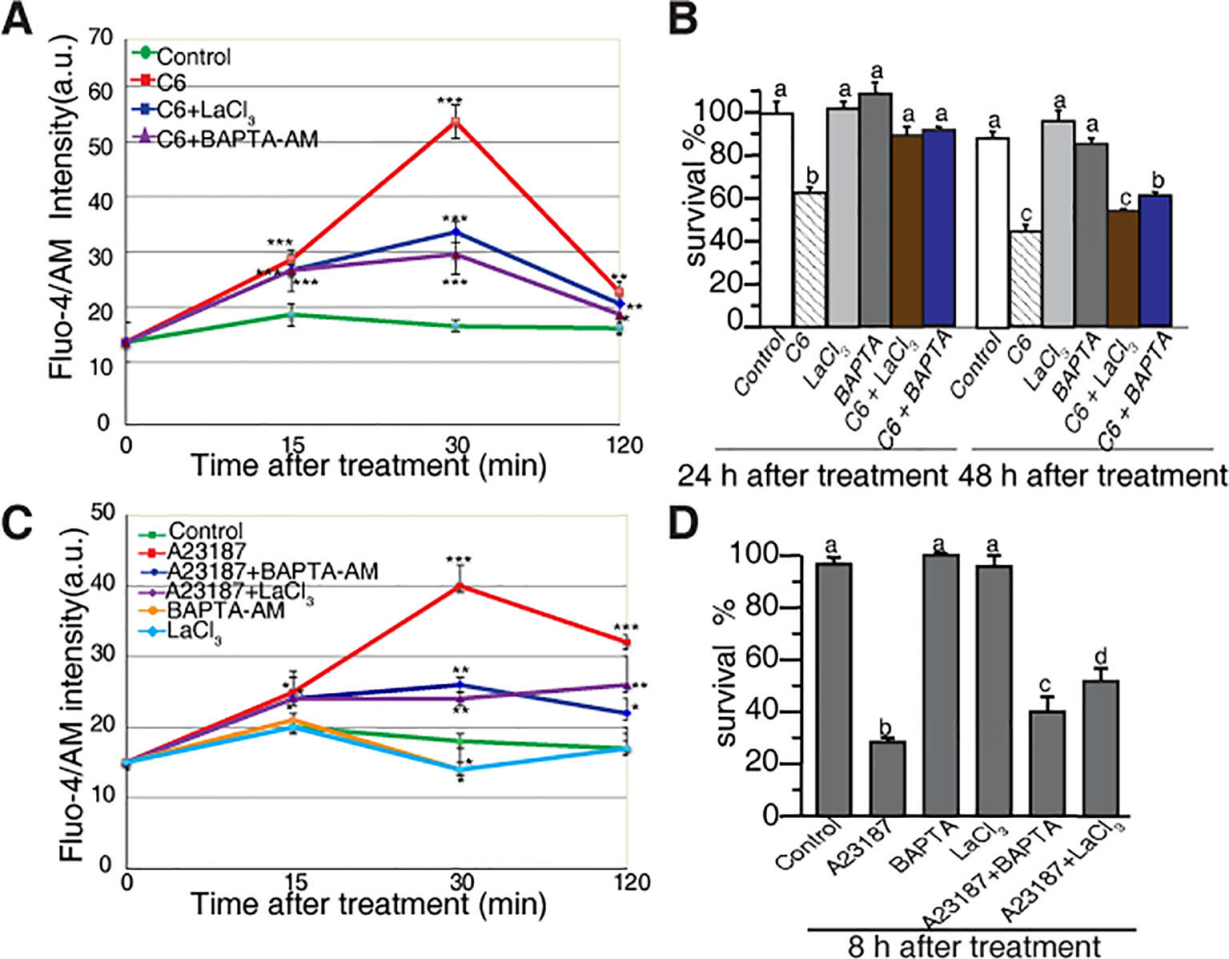
Calcium changes in rice protoplasts after C6 ceramide treatment. **(A)** The change in [Ca^2+^]_cyt_ in rice protoplasts treated with 0.1% EtOH (control), 100 μM C6 ceramide, 20 μM LaCl_3_, or 1 μM BAPTA/AM for 2 h. Calcium determined by Fluo-4/AM fluorescence intensity detected by flow cytometry. Cellular calcium measurements were repeated three times using independent samples. Asterisks show a significant difference from the 0.1% EtOH treatment based on PLSD (*p < 0.05, **p < 0.01, ***p < 0.001). Error bars indicate standard deviations. a. u. means arbitrary units. **(B)** Percentage of protoplast survival after 100 μM C6 ceramide or 1 μM BAPTA/AM 20μM LaCl_3_ treatments for 24 h or 48 h. FDA staining was used for detection of viable cells. This experiment was repeated three times using independent samples. Letters indicate that values differed based on in Fisher’s protected least significant difference (PLSD) test, a *post hoc* multiple t test (*p* < 0.05). Error bars indicate standard deviations. Control treatment was with 0.1% ethanol (the solvent for C6 ceramide). **(C)** The change in [Ca^2+^]_cyt_ in rice protoplasts treated with 0.1% EtOH (control); 10 μM A23187, 20 μM LaCl_3,_ 1 μM BAPTA/AM, 10 μM A23187 plus 1 μM BAPTA/AM, 10 μM A23187 plus 20 μM LaCl_3_ treatments for 2 h. Calcium determined by Fluo-4/AM in fluorescence intensity detected by flow cytometry. This experiment was repeated three times using independent samples. Asterisks show a significant difference from the 0.1%EtOH treatment based on PLSD (*p < 0.05, **p < 0.01, ***p < 0.001). Error bars indicate standard deviations. a. u. means arbitrary units. **(D)** The percentage of cell survival after 10 μM A23187, 20 μM LaCl_3_, 1 μM BAPTA/AM, 10 μM A23187 plus 1 μM BAPTA/AM and 10 μM A23187 plus 20 μM LaCl_3_ treatments for 8 h. This experiment was repeated three times using independent samples. Letters indicate that values differed based on Fisher’s protected least significant difference (PLSD) test, a *post hoc* multiple *t* test (*p* < 0.05). Error bars indicate standard deviations. Control treatment was with 0.1% ethanol (the solvent for C6 ceramide).

To investigate the early responses related to cell death in rice protoplasts treated with ceramides, we monitored the intracellular Ca^2+^ concentration based on the fluorescence of Fluo-4, using flow cytometry and confocal microscopy. The C6 ceramide treatment resulted in a rapid increase of intracellular calcium within 30 min, an effect that was partially abolished in the presence of the calcium channel blocker lanthanum (III) chloride (LaCl_3_) and the intracellular calcium chelator BAPTA-AM (**Figure 3A**). When treated with C2 ceramide the calcium change shows similar pattern with that of C6 ceramide treatment (**Supplemental Figure 2**).

We also evaluated the effect of free intracellular calcium on cell death induced by ceramides. Both LaCl_3_ and BAPTA-AM blocked C6 ceramide-induced cell death at 24 h. At 48 h, BAPTA-AM partially rescued cell death (**Figure 3B**). These observations indicated that in rice, intracellular calcium plays an important role in ceramide-induced protoplast cell death.

To further examine the role of calcium influx, we used the calcium ionophore A23187 to treat rice cells. As shown in **Figure 3C**, A23187 rapidly enhanced Fluo-4 signals within 15 min. LaCl_3_ and BAPTA-AM partially blocked intracellular calcium changes and partially rescued A23187-induced cell death (**Figure 3D**). These data again highlight the involvement of intracellular Ca^2+^ in the early phase of ceramide-induced PCD.

### ROS Accumulation in Ceramide-Induced Rice PCD

**Figure 4:**
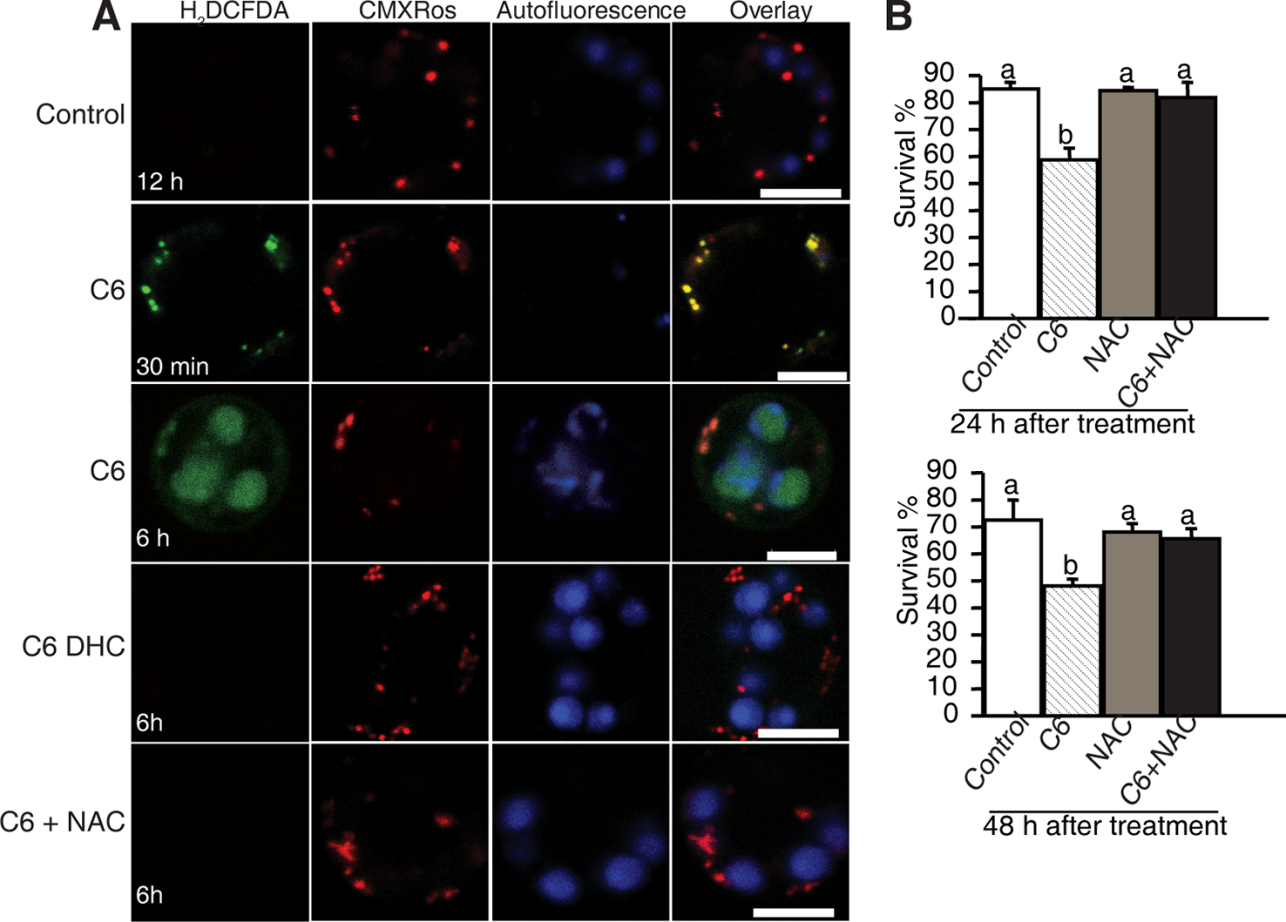
ROS detection after ceramide treatments. **(A)** Confocal micrographs of ROS generation in ceramide-treated cells. Protoplasts from 10-day-old rice seedlings were treated with 100 μM C6 ceramide 100 μM C6 DHC, 5 μg/ml ROS scavenger NAC or 0.1% EtOH (solvent control) for 6 h and then stained with CM-H2DCFDA (green) and CMXRos (red). Images were taken by confocal microscopy. The red fluorescence indicates mitochondria (beam-splitter: MBS 458/561; emission filter: 568–691). Autofluorescence (blue) indicates chloroplasts (beam-splitter: MBS 488/561/633; emission filter: 647–722). This experiment was repeated three times using independent samples. Bar = 5 μm. **(B)** The survive rate of protoplasts treated with different reagents. Rice protoplasts were treated with 0.1% EtOH (solvent control), 100 μM C6 ceramide, 5μg/ml NAC, or NAC + 100 μM C6 ceramide for the indicated times under light. The experiment was repeated three times using independent samples. Letters indicate that values differed based on PLSD (*p* < 0.05). Error bars indicate standard deviations.

To investigate the early responses and the subcellular localization of signals related to cell death in rice protoplasts treated with ceramides, we used CM-H_2_DCFDA (DCF) as a marker to monitor intracellular ROS and MitoTracker Red (CMXRos) to stain mitochondria. As early as 30 min after C6 ceramide treatment, DCF staining detected strong ROS signals (**Figure 4A**, horizontal second panel). Double staining with MitoTracker Red revealed that most DCF-stained regions colocalized with mitochondria, suggesting that mitochondrial-derived ROS production is an early event during ceramide-induced cell death. We also noticed that at 6 h after C6 treatment, ROS mainly accumulated on chloroplasts (**Figure 4A**, the third horizontal panel). No signals were observed in DCF staining in the control protoplasts not treated with C6 ceramide (**Figure 4A**) or C6 dihydroceramide (C6 DHC) along a time course (**Figure 4A**, fourth horizontal panels).

To examine the effect of ROS scavengers on ceramide-induced ROS production and cell death, rice protoplasts were incubated with the free radical scavenger N-acetyl-LL-cysteine (NAC). As shown in **Figure 4**, NAC completely depleted ROS accumulation (**Figure 4A**, the fifth horizontal panel and **Supplemental Figure 3**) and blocked cell death induced by ceramides (**Figure 4B**), indicating that ROS play a crucial role in C6 ceramide-induced PCD.

### Ceramide Treatment Induced Mitochondrial Dysfunction but Not Cytochrome *c* Release

**Figure 5:**
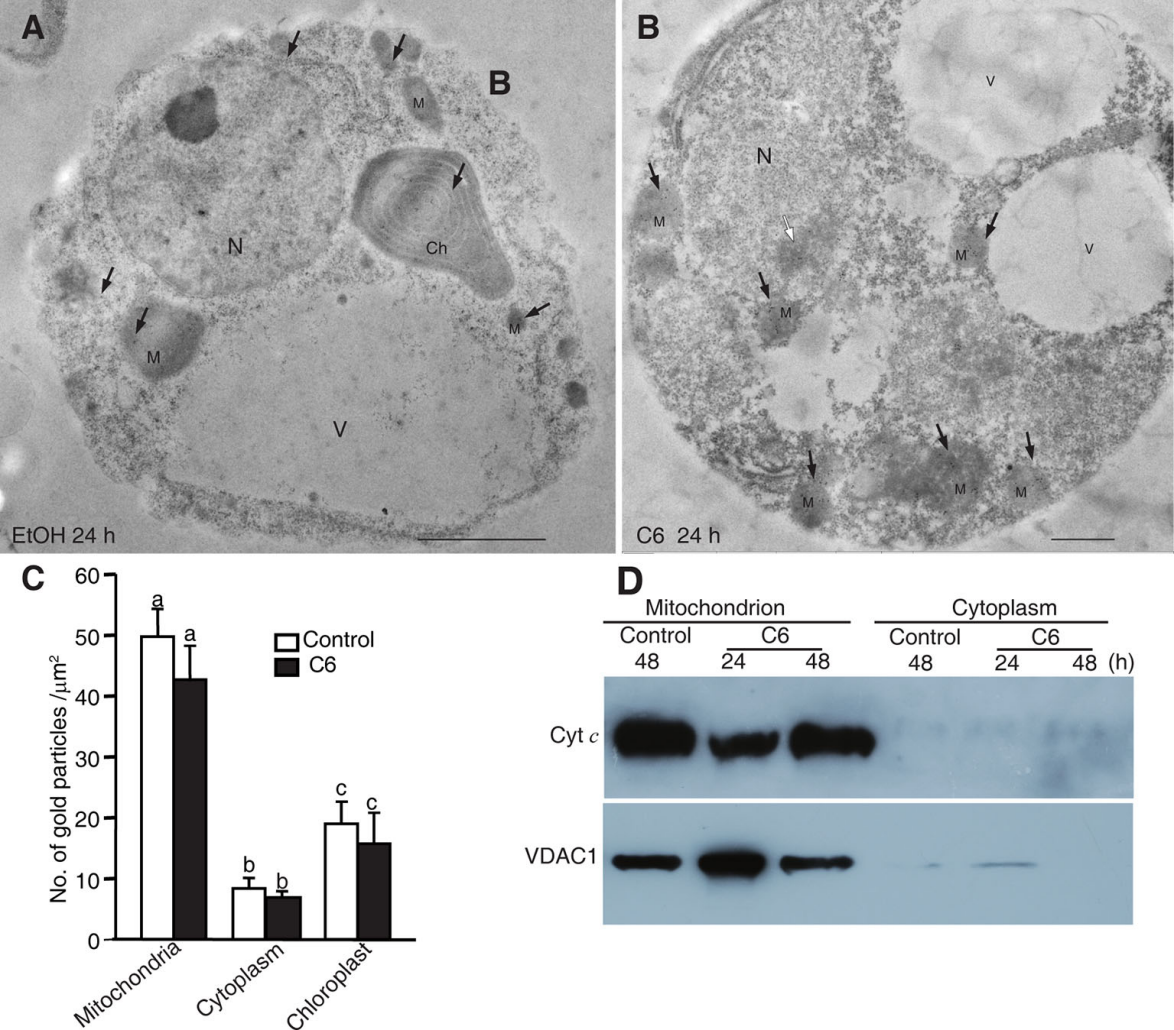
Localization of Cytochrome *c* after C6 ceramide treatment. **(A)** Immunolocalization of Cyt *c* in protoplasts after 0.1% EtOH treatment for 24 h. Black arrowheads indicate the particles of immunogold-labeled Cyt *c*. Ch, chloroplast; M, mitochondrion; N, nucleus; V, vacuole. Bars = 1 μm. **(B)** Immunolocalization of Cyt *c* in protoplast after 100 μM C6 ceramide treatment for 24 h. Black arrowheads indicate the particles of immunogold-labeled Cyt *c*. White arrowhead indicates the nuclei with apparently condensed chromatin. Ch, chloroplast; M, mitochondrion. V, vacuole. Bars = 1 μm. **(C)** The histogram shows the quantification of gold particles. At least 40 cells in each treatment were observed for statistical analysis of gold particles. Letters indicate that values were different based on PLSD test. Note no difference was shown between control and C6 ceramide treatments. **(D)** Cyt *c* measured by western blot. Cells were treated with 100 μM C6 ceramide or solvent control (0.1% EtOH) for 48 h and blotted with anti-Cytochrome *c* monoclonal antibodies.

Release of Cyt *c* from mitochondria into the cytosol has been reported to occur in ceramide-induced apoptosis in mammalian cells. Previously, we found that ceramide could induce PCD, as shown by mitochondrial membrane potential loss and the presence of DNA laddering (Bi et al., 2011). To investigate whether Cyt *c* release occurs in ceramide-induced PCD, we examined Cyt *c* localization in ceramide-treated cells by immuno-electron microscopy. We found that Cyt *c* mainly localized in mitochondria. However, Cyt *c* also localized in the chloroplasts and the cytosol after C6 ceramide treatment, similar to control treatments (**Figures 5A, B**). Statistical analysis of the gold particle density showed that no significant change occurred either in mitochondria or the diffuse localization in the cytoplasm, compared with control treatment (**Figure 5C**). The data indicated that Cyt *c* remained in the mitochondria during ceramide-induced cell death and Cyt *c* release is not related to chromatin condensation and cell death.

To confirm the Cyt *c* localization, we made mitochondrial and cytosolic fractions from rice protoplasts. Immunoblotting with anti-Cyt *c* antibodies was used to detect Cyt *c* in the mitochondrial and cytosolic fractions at different time points after C6 ceramide treatment. As shown in **Figure 5D**, Cyt *c* was in the mitochondria at 24 and 48 h after C6 ceramide treatments, whereas no Cyt *c* was in the cytosolic fractions in control or ceramide-treated protoplasts. Voltage dependent anion channel (VDAC1) was used as a mitochondrial marker.

In addition, we further investigated the Cyt *c* distribution using immunofluorescence staining. At ceramide treatment, although the mitochondria were swelling, the Cyt *c* signals strictly localized in the mitochondria, similar to the control cells (**Supplemental Figure 4**). Therefore, three different experiments regarding Cyt *c* localization after C6 ceramide treatment showed that Cyt *c* was not released from the mitochondria into the cytosol in ceramide-induced rice cell death.

### Involvement of VDAC and ATP Loss in Ceramide-Induced PCD

**Figure 6:**
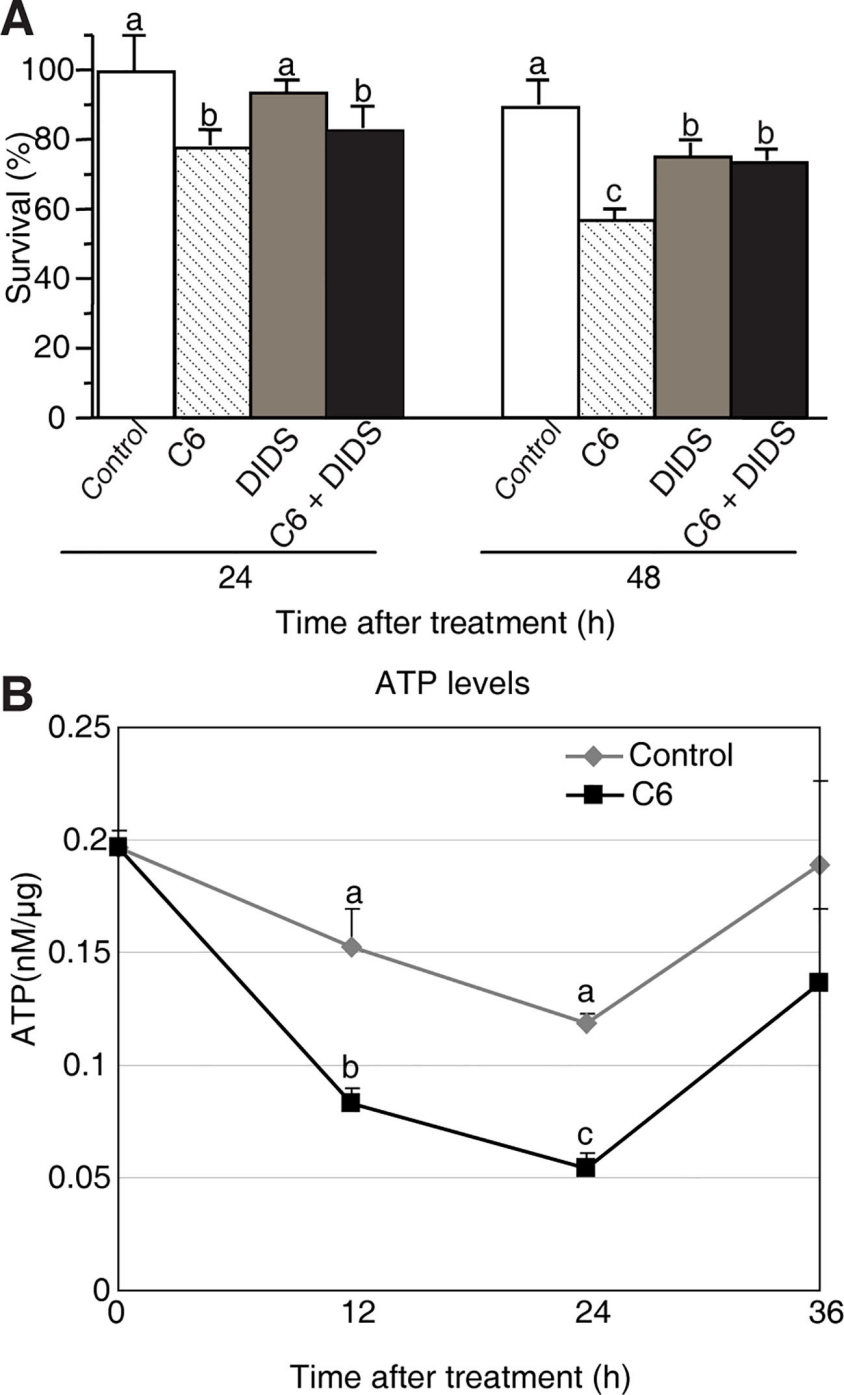
Mitochondrial dysfunction induced by C6 ceramide treatments. **(A)** The survive rate of protoplasts treated with different reagents. Rice protoplasts were treated with control (0.1% ethanol plus 0.1% DMSO), 100 μM C6 ceramide, 1 μM DIDS, or 1 μM DIDS plus 100 μM C6 ceramide for the indicated times. The experiment was repeated three times using independent samples. Letters indicate that values differed based on PLSD (*p* < 0.05). Error bars indicate standard deviations. Control treatment was with 0.1% ethanol (the solvent for C6) plus 0.1% DMSO (the solvent for DIDS). **(B)** ATP levels were detected by using a luminometer at different time points. Protoplasts were treated with 100 μM C6 ceramide or 0.1% EtOH (as a control). ATP was measured as described in the *Methods* at the indicated times. The experimental points represent mean values from three replicate experiments. Letters indicate that values differed based on PLSD (*p* < 0.05). Error bars indicate standard deviations.

Since our results showed that Cyt *c* release is not a feature of ceramide-induced rice cell death, we next tested whether the permeability transition pore (PTP) was open. To investigate the involvement of the PTP complex, we used the VDAC inhibitor 4, 49-diisothiocyanatostilbene-2, 29-disulfonic acid (DIDS), a nonselective inhibitor of anion exchangers and channels (Keinan et al., 2010). We found high concentrations of DIDS (10–100 μM) had a dramatic effect on cell death in rice protoplasts (**Supplemental Figure 5A**), whereas low concentrations of DIDs (1 μM) had no effect on cell death; therefore, we used 1 μM DIDS in the following experiments (**Supplemental Figure 5B**). We first pretreated protoplasts with DIDS for 1 h, then added C6 ceramide and incubated for 24–48 h. DIDs partially rescued the C6 ceramide-induced cell death at 48 h (**Figure 6A**), suggesting that the VDAC was important for ceramide-induced PCD.

It has been reported that ATP is required to carry out PCD (Sun et al., 2012). To further test the cell death that occurs in C6 ceramide-treated protoplasts, we monitored ATP levels by using a luminometer at different time points. The total ATP level decreased by approximately 50% at 12 h after ceramide treatment, compared with control cells. At 24 h the ATP level was the lowest, and then it significantly increased at 36 h (**Figure 6B**). The data indicated that C6 ceramide treatment of rice cells affects mitochondrial function and, as a consequence, causes a decrease in the rate of ATP synthesis.

### Caspase-Like Protease Activity During PCD

**Figure 7:**
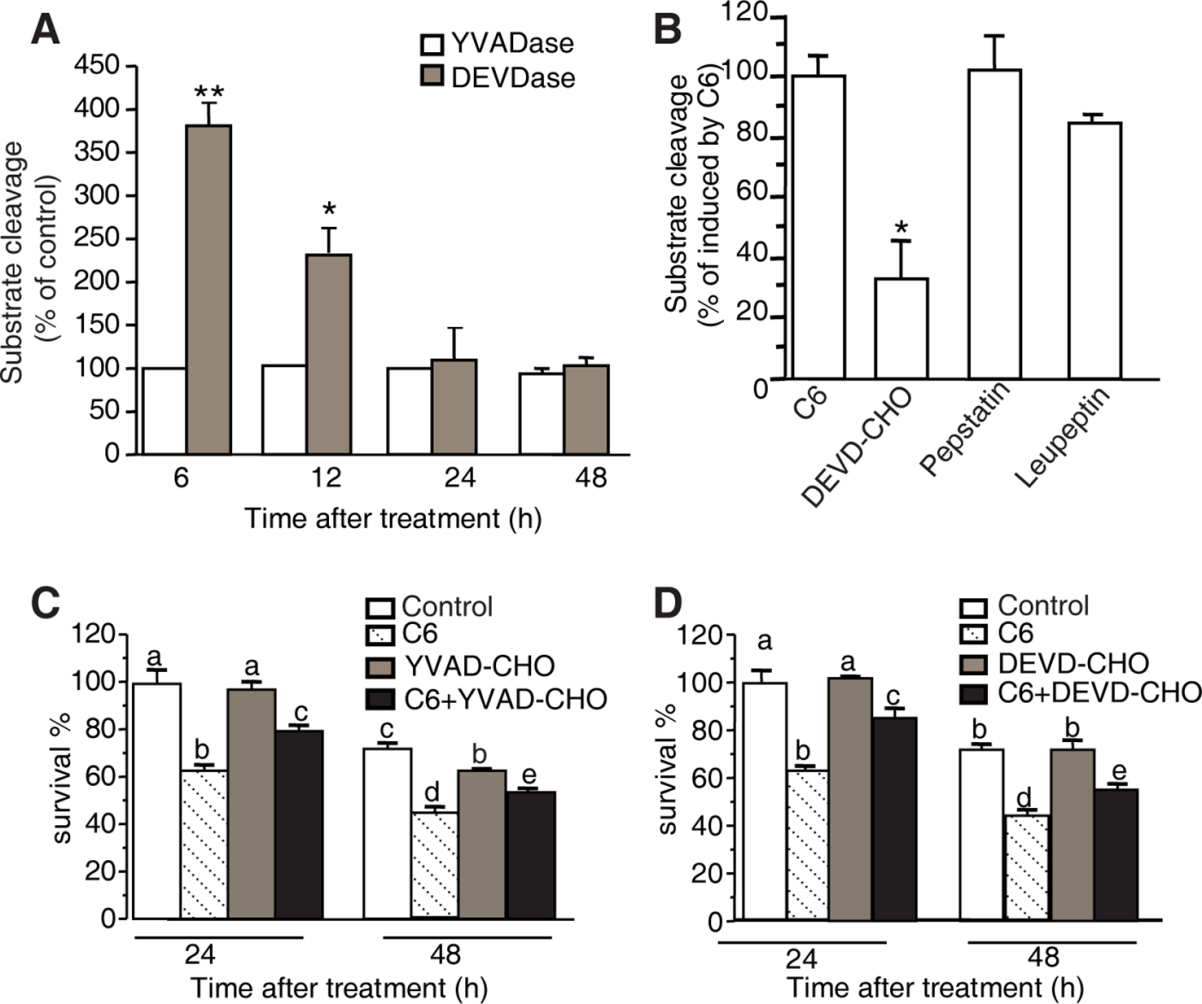
YVADase and DEVDase activity measurements after C6 ceramide treatments. **(A)** Substrate specificity of C6 ceramide-induced caspase-1 (YVADase) and-3 (DEVDase) like activities in rice protoplast extracts. Caspase-like activities were assayed by measuring the fluorescence intensity of the cleaved specific caspase-1 substrate Ac-YVAD-AMC and caspase-3 substrate Ac-DEVD-AMC. The assay was repeated three times with similar results. Letters indicate that values are different based on (*p* < 0.05). Bars show standard deviations. **(B)** Effect of different protease inhibitors on DEVDase activity after C6 ceramide treatment for 6 h. The fluorescence units were measured and are expressed as percentage of the enzyme activity of solvent-treated protoplasts (100%). Protoplast extracts were pre-incubated with 50 μM Ac-DEVD-CHO, 200 μM pepstatin A, or 100 μM leupeptin for 1 h before adding Ac-DEVD-AMC. Error bars indicate ± SE from three technical replicates. The assay was repeated two times using independent samples. Asterisks show a significant difference from the enzyme activity of solvent-treated protoplasts Student’s *t*-test (*p < 0.05). **(C, D)** Effect of caspase-specific inhibitors on cell viability. Ac-YVAD-CHO **(C)** and Ac-DEVD-CHO **(D)** partially decrease the C6 ceramide-induced cell death. Protoplasts were pretreated with 20 μM caspase specific inhibitors Ac-YVAD-CHO or Ac-DEVD-CHO for 1 h and then treated with 100 μM C6 ceramide for 24 or 48 h. Degree of cell death was estimated by FDA staining. The assay was repeated three times with similar results. Letters indicate that values are different based on PLSD (*p* < 0.05). Bars show standard deviations.

Cellular caspases belong to a highly conserved family of cysteine proteases that cleave aspartate residues of caspases and functions as major players in the execution of apoptosis in animals. Recent work has identified several caspase-like activities in plant PCD (Cai et al., 2018), such as caspase-1 and caspase-3. To examine these caspases in our system, we used fluorogenic AMC substrates to measure caspase activities in protoplasts after C6 ceramide treatment, testing caspase-1 substrates (Ac-YVAD-AMC) and caspase-3 substrates (Ac-DEVD-AMC). As shown in **Figure 7A**, at 6 h of C6 ceramide treatment, caspase-3 like protease activity increased about four-fold compared with control. The level of caspase-3 like protease activity returned to control levels at 24 h. In contrast, no significantly elevated caspase-1 like protease activity was detected in the C6 ceramide-treated extracts (**Figure 7A**).

To identify the specific proteolytic enzyme activity in extracts of C6 ceramide-treated protoplasts, we pre-incubated the protoplast extracts with caspase-specific inhibitors (Ac-DEVD-CHO and Ac-YVAD-CHO) and general protease inhibitors (pepstatin A and leupeptin) for 1 h before adding the fluorogenic substrate Ac-DEVD-AMC. The caspase-3 like protease activity induced by C6 ceramide treatment was inhibited by 50 μM Ac-DEVD-CHO, whereas other protease inhibitors such as pepstatin A and leupeptin had no significant effect on the caspase-3-like protease activity (**Figure 7B**).

Furthermore, we measured viability in protoplasts pretreated with the caspase-specific inhibitors Ac-DEVD-CHO or Ac-YVAD-CHO for 1 h before C6 ceramide treatments. Ac-DEVD-CHO and Ac-YVAD-CHO decreased C6 ceramide-induced cell death and this rescue lasted for 48 h after treatment (**Figures 7C, D**).

## Discussion

In this report, we demonstrated that ceramide treatment stimulates ceramide accumulation in mitochondria and stimulates ROS production, calcium elevation in the cytosol, and caspase-3 like protease activity, but does not involve the translocation of Cyt *c* from the mitochondria to the cytosol. The present results provide new evidence that ceramide-induced PCD may occur by different mechanisms in Arabidopsis and rice.

Plant PCD occurs during development as well as in response to environmental and biotic stimuli (Van Hautegem et al., 2015; Dickman et al., 2017). Numerous observations suggest that ceramide can modulate PCD in plants (Liang et al., 2003; Bi et al., 2014; Li et al., 2016). However, the ceramide-induced signaling pathway leading to PCD remains largely unknown in rice. Based on ultrastructural examination, we observed condensed chromatin in C6 ceramide-treated rice cells. We also showed that exogenous C6 ceramide increased intracellular levels of LCB and ceramide. The increased cellular levels of endogenous ceramides may be synthesized *via* a sphingosine-recycling pathway (Ogretmen et al., 2002). Indeed, previous studies have suggested a direct relationship between exogenous and endogenous ceramides. For example, cell-permeant exogenous ceramides can trigger neutral sphingomyelinase activation, sphingomyelin hydrolysis, and endogenous ceramide generation (Jaffrézou et al., 1998). Thus, there appear to be multiple mechanisms by which exogenous ceramides influence endogenous ceramide metabolism. We cannot rule out that LCBs also play a role in C6-induced cell death, as LCBs are important second messengers for PCD. Nevertheless, our current data suggest that endogenous ceramide accumulation is one of the main events that occur after C6 ceramide treatment. According to our observation, the increased ceramide induced by C6 treatments mostly accumulated in mitochondria, as detected by immuno-labeling with anti-ceramide antibodies. Combined with our previous result that DNA laddering occurs after C6 ceramide treatment (Bi et al., 2011), we find that ceramide can induce PCD in rice, a phenomenon that may be related to mitochondrial dysfunction (Huang et al., 2016).

Ceramide, a key compound of the sphingolipid metabolic pathway, provoked PCD *via* elevation of cytosolic calcium in Arabidopsis (Townley et al., 2005). Similarly, in tobacco cells, external application of dihydroxy-LCB causes an immediate dose-dependent elevation of cellular free calcium within the first minute in the cytosol and ten minutes later in the nucleus, followed by H_2_O_2_ accumulation and cell death (Lachaud et al., 2010; Lachaud et al., 2011). In our results, C2 and C6 ceramides elicited a dramatic increase in calcium levels within 15 min, prior to cell death. La^3+^ and Ca^2+^ have very similar structure and ion radius and La^3+^ may replace the Ca^2+^ binding site (Aussel et al., 1996). So, LaCl_3_ could be used as calcium channel blocker to decreased the presumed cytosolic Ca^2+^ level. The intracellular calcium chelator BAPTA-AM can also partially abolish this elevation of calcium levels and rescued ceramide-induced cell death. These data indicated that intracellular calcium plays an important role in ceramide-induced PCD in rice. Moreover, treatment of rice protoplasts with C6 ceramide elicits an increase in ROS. The ROS scavenger NAC prevented ROS accumulation and blocked cell death induced by ceramides, suggesting that ROS participate in ceramide-induced PCD, consistent with previous reports (Yao et al., 2004; Bi et al., 2014).

Caspases are key regulators of apoptosis (Riedl and Salvesen, 2007). Although plant genomes have no sequences that have been identified as encoding caspases, caspase inhibitors can suppress PCD in plants, similar to results in animal systems (Sueldo and van der Hoorn, 2017). Here, we found that the activity of caspase-3 like proteases increased significantly in C6 ceramide-treated rice protoplasts and a caspase-3 specific inhibitor can rescue C6 ceramide-induced cell death. Ac-YVAD-CHO could also decrease C6 ceramide-induced cell death though no significantly elevated caspase-1 like protease activity was detected in the C6 ceramide-treated extracts. Ac-YVAD-CHO may inhibit other caspase-like proteases beside caspase-1 in rice. Take these results together, C6 ceramide-induced PCD requires caspase activity. However, ceramide-induced PCD in rice did not involve the translocation of Cyt *c* from the mitochondria to the cytosol, which differs from ceramide-induced cell death in Arabidopsis (Yao et al., 2004). In this paper, we used three different experiments (immuno-electron microscopy, western blot, immunofluorescence staining) to confirm that no Cyt *c* was detected in cytosol during C6 induced PCD in rice. Interestingly, the cytochrome c levels in mitochondria alternated after C6 treatments. We think there were several possibilities. The first possibility was that we could not measure protoplast dosage in different times. The second possibility was that the released cytochrome c was ubiquitinated and degraded through sphingolipid-dependent activation of the proteasome (Kroesen et al., 2003; Gama et al., 2014). The third possibility was cytochrome *c* was degraded *via* caspase 3-like proteases increased by C6 treatment (Vacca et al., 2006).

Our observation of ceramide accumulation in the mitochondria and ATP loss after ceramide treatment provides potential insight into the mechanism of ceramide-induced PCD. Moreover, VDAC inhibition can rescue ceramide-induced cell death. In animal cells, ceramide causes the release of Ca^2+^ from the endoplasmic reticulum and thus Ca^2+^ increased both in the cytosol and mitochondria, which leads to opening of the PTP and ATP depletion, as well as the activation of Ca^2+^-dependent proteases (Pinton et al., 2001; Giorgi et al., 2008). Moreover, intracellular Ca^2+^ positively regulates the activity of the nuclear transcription factor-kB (NF-kB), which can regulate the activity of caspase-3 (Sun and Carpenter, 1998). Our hypothesis for ceramide-induced cell death of rice protoplasts is that following ROS accumulation and oxidative damage of cell membranes, calcium or caspase-like enzymes escape from the broken organelles, possibly resulting in PCD (Carraro and Bernardi, 2016; Xiao G. et al., 2018).

## Data Availability Statement

All data supporting the conclusions of this article are provided within the article and its additional files (**Additional file 1: Figure S1**, **Additional file 2: Figure S2**, **Additional file 3: Figure S3**, **Additional file 4: Figure S4**, **Additional file 5: Figure S5**).

## Author Contributions

NY and Q-FZ conceived and designed experiments. Q-FZ, JL, F-CB, ZL, Z-YC, L-YW and L-QH performed the experiments. Q-FZ and JL analyzed the data. NY, JL, and Q-FZ wrote the article. All authors have discussed the results and contributed to the drafting of the manuscript. All authors read and approved the final manuscript.

## Funding

This work was supported by the National Natural Science Foundation of China (31700221, 31771357, 31570255), Natural Science Foundation of Guang dong Province (2017A030311005) and Fundamental Research Funds for the Central Universities (18lgpy51).

## Conflict of Interest

The authors declare that the research was conducted in the absence of any commercial or financial relationships that could be construed as a potential conflict of interest.
