# Supplementary figures and images for: Ceramide-Induced Cell Death Depends on Calcium and Caspase-Like Activity in Rice
Source: Front Plant Sci. 2020 Feb 26;11:145. doi: 10.3389/fpls.2020.00145 (PMC7054224; doi:10.3389/fpls.2020.00145)

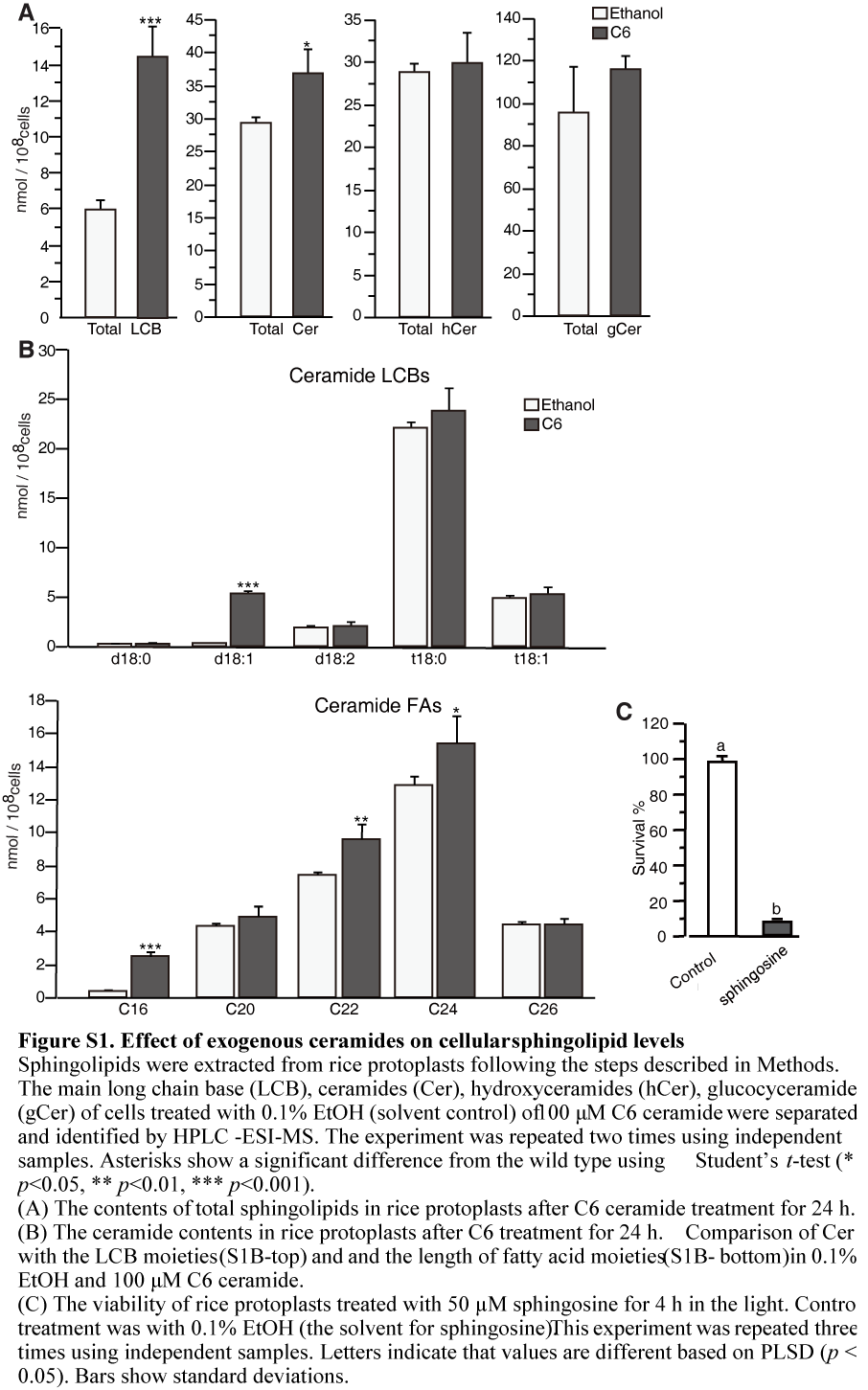

Supplement: Supplementary file 1 [file Image_1.tif]

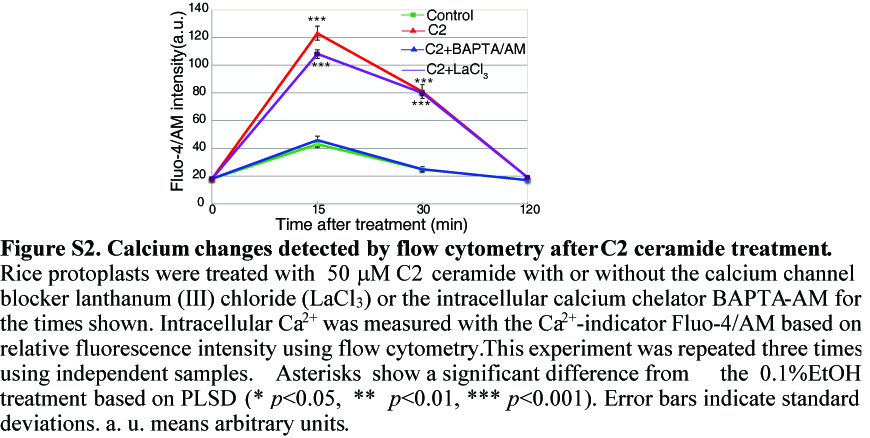

Supplement: Supplementary file 2 [file Image_2.tif]

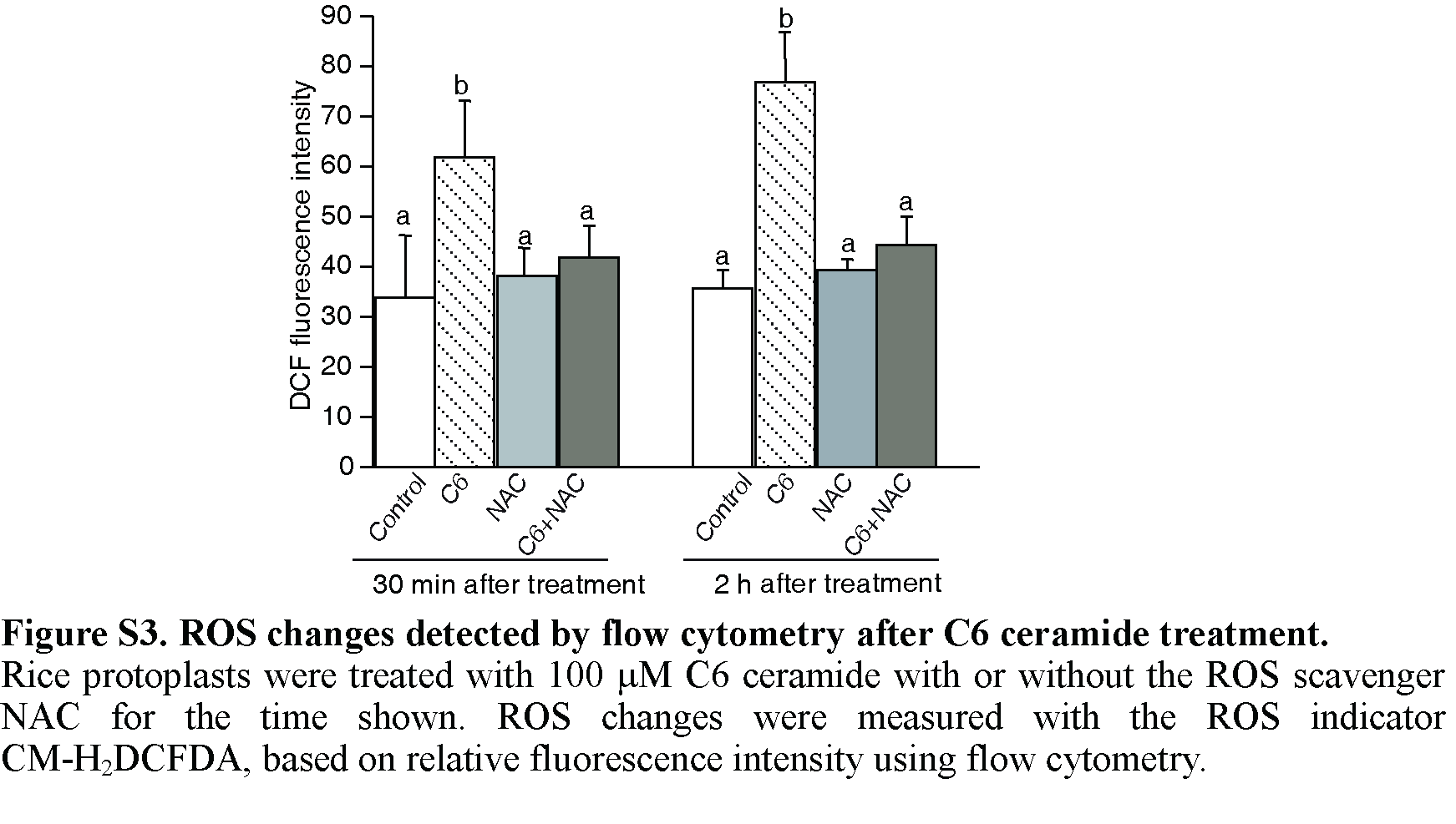

Supplement: Supplementary file 3 [file Image_3.tif]

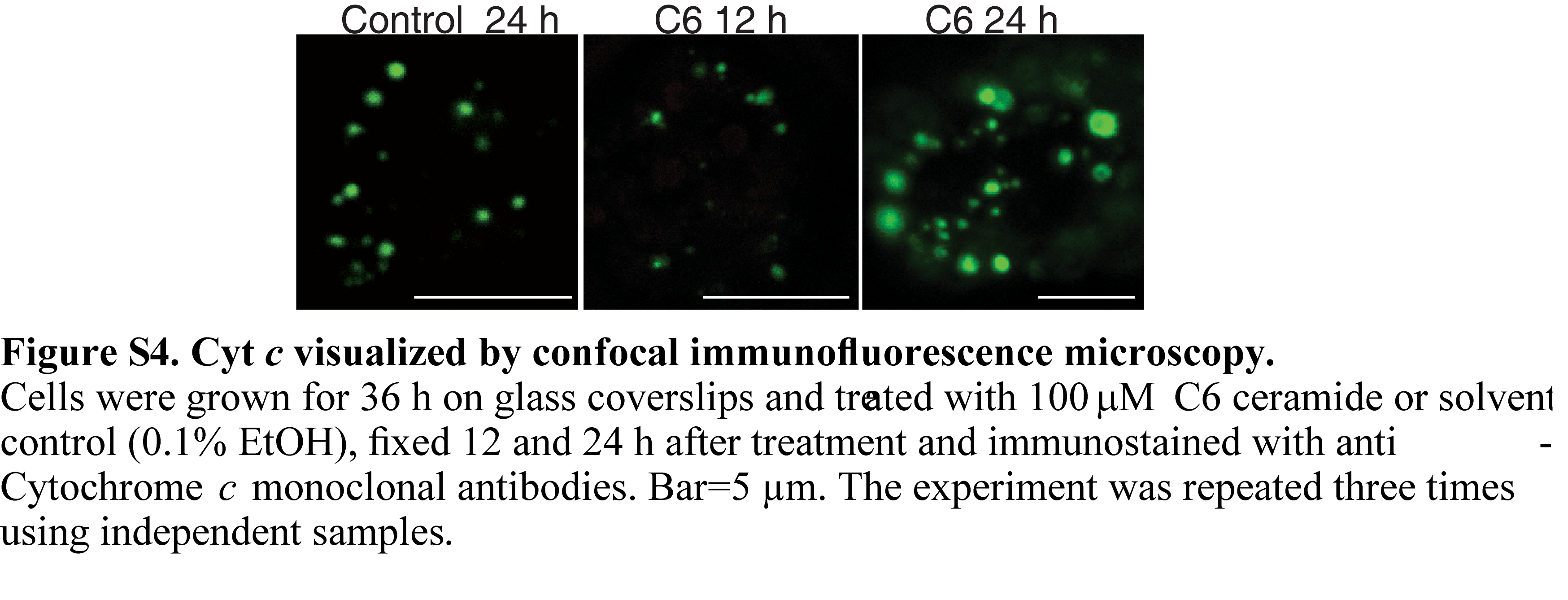

Supplement: Supplementary file 4 [file Image_4.tif]

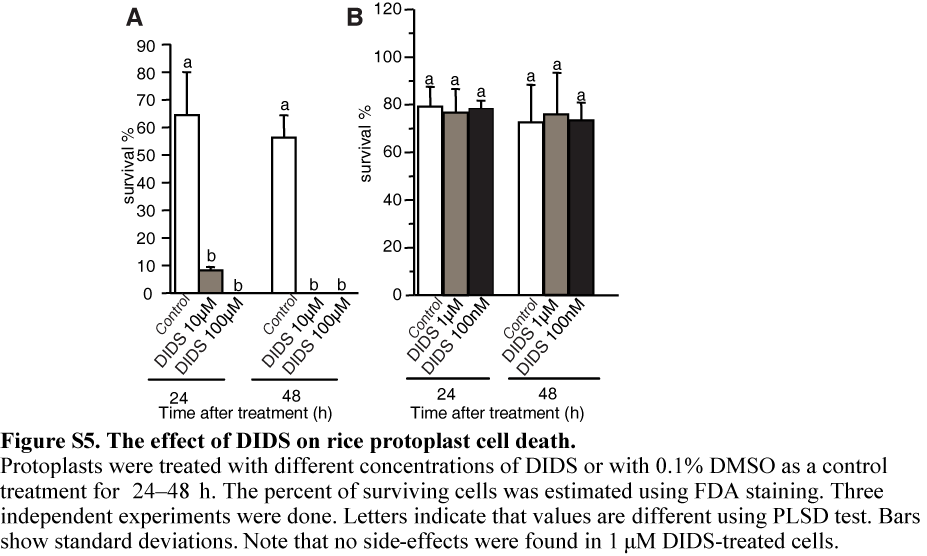

Supplement: Supplementary file 5 [file Image_5.tif]
